# Potential profiling of social alienation in older female patients with stress urinary incontinence

**DOI:** 10.3389/fpubh.2024.1496539

**Published:** 2025-01-15

**Authors:** Yakun Li, Hongxia Wang

**Affiliations:** School of Nursing, Jinzhou Medical University, Jinzhou, China

**Keywords:** older people, stress urinary incontinence patients, social alienation, latent profile analysis, Influencing factors

## Abstract

**Background:**

With the global trend of aging, stress urinary incontinence is becoming more common in older adults, which may have some impact on patients' quality of life. Social alienation can generate negative emotions such as anxiety, depression, loneliness, and morbid stigma, and reduce patients' quality of life. However, the current status of social alienation is different among different older adult female patients with stress urinary incontinence. Therefore, this study categorizes older adult female stress urinary incontinence patients through potential analysis to understand the category characteristics of social alienation level of older adult female stress urinary incontinence patients, and explores the influencing factors of social alienation level of different categories of older adult female stress urinary incontinence patients, which can provide a reference to personalized intervention programs for the characteristics of social alienation of older adult female stress urinary incontinence in the future.

**Method:**

A convenience sampling method was used to select 365 cases of older adult female stress urinary incontinence patients from March 2023 to April 2024 in three communities in Jinzhou City. The General Information Questionnaire, the Family Care Index Scale, the Toronto Alexithymia Scale, and the General Alienation Scale were used to conduct the survey.

**Results:**

A total of 365 respondents were included, and three potential categories of social alienation were finally identified, namely, low social alienation (29.0%), medium social alienation-self alienation (49.4%), and high social alienation (21.6%). The results of multifactorial logistic regression analysis showed that occupational status, marital status, whether living alone, place of residence, BMI, whether other chronic diseases, level of narrative disorders, and level of family care were the influencing factors of social alienation in older adult female patients with stress urinary incontinence (*P* < 0.05).

**Conclusion:**

The social alienation of older adult female patients with stress urinary incontinence is characterized by a significant number of categories, and healthcare professionals can identify the characteristics and influencing factors of each category at an early stage, which can provide a basis for the development of targeted clinical interventions to help patients reduce the level of social alienation.

## Introduction

Stress urinary incontinence (SUI), usually refers to the involuntary flow of urine from the external urethral orifice of the body as a result of increased abdominal pressure (e.g., laughing, coughing, sneezing, or exercising) (1). SUI, as a common and chronic condition in women, affects people of all ages and increases with age (2). According to the data of the seventh national census of the National Bureau of Statistics in 2020, China's older adult population aged 60 years and above in China has increased from 13.26% to 18.70% in the past 10 years. By 2035, China's older adult population aged 60 years and above will break through 400 million, and the proportion of the total population will be more than 30% entering a heavily aging society. This demonstrates the significant change in the aging demographic structure of China's society. And with the deepening process of population aging, the incidence of SUI is also growing (3). Epidemiologic surveys have shown (4), that 25–45% of women worldwide have varying degrees of urinary incontinence, and the prevalence of SUI in adult women in China is 18.9%, while the prevalence of SUI in older adult women is as high as 30.5%. Although this disease does not lead to organic lesions, frequent leakage of urine can lead to problems such as urinary system inflammation, bedsores, eczema, etc., accompanied by odors and adverse life experiences caused by urine leakage, which not only seriously affects the daily work and life of the patients, and has a long-lasting adverse effect on the health and quality of life of older adult women, but also produces negative emotions such as anxiety, depression, loneliness, and morbid stigma, and in the process of socialization behaviors such as closure, avoidance, and withdrawal, resulting in a sense of social alienation (5, 6).

Social alienation refers to the individual in the process of social interaction, social will is not satisfied, cannot interact well with the outside world, and then alienated from others and social relations or even isolated and rejected by others, resulting in loneliness, rejection and other negative emotions and behaviors (7, 8). In China, the social alienation of older adult women with stress urinary incontinence is still at a high level (9). Most of the older adults with stress incontinence are more likely to have various physical and psychological problems due to the decline of their physical functions and weakening of their ability to take care of themselves, as well as the worsening symptoms of incontinence. They may regard incontinence as a defect of the body and worry about the negative evaluation of themselves by others, and therefore are more likely to actively alienate themselves from the social circle, which leads to a high level of social alienation. In the Chinese culture of implicit and introverted, female SUI patients are more conservative in their cognition and attitudes, and they often feel ashamed of their disease, shy away from seeking early medical treatment, and are unwilling to express their true feelings with others (10), which further aggravates the sense of social alienation. Studies have shown that reducing the level of social alienation can improve the health status and quality of life of patients and reduce the incidence of complications (11).

Currently, most of the studies on social alienation in older adult women with SUI focus on the “variable-centered” perspective, using scale scores to describe the level of social alienation and the factors affecting the patient's social alienation, and lack of individual-centered research on the heterogeneity of the population, which restricts the ability to make precise interventions. Different older adult women with SUI have different social alienation problems, making it difficult to provide guidance through a uniform program and lacking targeted intervention programs for different populations. Therefore, a categorical analysis of the heterogeneity of the population's social alienation ability can be considered to clarify the characteristics and influencing factors of social detachment in different groups, and to facilitate targeted intervention programs at a later stage.

Latent profile analysis (LPA) is an individual-centered statistical analysis method, which explains the associations between external continuous variables through latent category variables and realizes local independence between exogenous variables (12). This study divides the available population into several categories through potential category analysis, revealing the heterogeneity of the target population (13). And multiple logistic regression analysis was used to explore the influencing factors of social detachment in SUI patients with different profiles, which is conducive to the development of targeted intervention programs according to the different needs of older adult patients in the later stages of their lives and improve their quality of life. The ABC-X stress theory model provides the theoretical basis for this study, which was proposed by Reuben Hill, the father of family stress theory, and contains a total of A, B, C, and X four basic Determinants: A represents the stressor event, which refers to any internal and external environmental stimuli that can cause individuals to have stress reactions, i.e., SUI is a common stressor event; B represents the resources, i.e., the family care of the patients in this study. Factor C represents the cognition or perception of the stressful event, which is the individual's subjective cognition of the stressful event, and there is inconsistency in different individuals' perceptions of it, i.e., the patients in this paper hold a narrative barrier mentality toward the SUI event. factor X is the outcome, i.e., the degree of harm caused by the stress or crisis to the individual, i.e., the sense of social detachment caused by the frequent SUI events, and the development of the model has been relatively mature, and has been widely applied in many professional fields been widely applied (14, 15).

This study used potential profile analysis to explore the potential categories of social alienation in stress urinary incontinence in older women, analyze their influencing factors, and provide a new basis for reducing the level of social alienation in stress urinary incontinence in older women, and for developing effective and scientifically individualized interventions.

## Method

### Study design and participants

From March 2023 to April 2024, a total of older adult women from three communities (Guta, Linghe, and Taihe districts) in Jinzhou City, Liaoning Province, were selected for the study using the convenience sampling method. Those who met the inclusion criteria in the three communities were included in the study. Inclusion criteria: (1) those who met the diagnostic criteria of the International Continence Advisory Committee Urinary Incontinence Questionnaire Short Form (ICI-Q-SF) (15) and were determined to have comorbid stress urinary incontinence symptoms; (2) females ≥60 years of age; and (3) informed consent and voluntary participants. Exclusion criteria:

(1) those with severe psychosomatic diseases or dementia; (2) those with a complete inability to communicate, such as aphasia or hearing dysfunction; (3) those with severe cardiovascular, cerebrovascular, hepatic, renal, and other chronic diseases. Participants had signed an informed consent form, strictly followed the voluntary principle, and kept the data anonymous and confidential. This study was approved by the Ethics Committee of Jinzhou Medical University under its approval number (JZMULL-2023109).

According to Kendall's principle (16): the sample size is 10–20 times of the independent variables, the independent variables in this study were 20, considering 20% of invalid questionnaires, the sample size was calculated to be 240–480 cases, and 365 older adult female patients with stress urinary incontinence were actually included in this study.

### Instruments

#### General information questionnaire

Based on a review of the literature, a general information questionnaire was designed based on the patient's disease status and experts. It included the participants' age, BMI, education level, occupational status, mode of residence, living environment, per capita monthly household income, and number of other chronic diseases combined.

#### Family care index scale (APGAR)

The family care index scale was used to assess the family functioning and caring of the respondents. The scale covers five dimensions: family adjustment, cooperation, adulthood, affectivity and closeness, and uses a 3-point Likert scale with a total score ranging from 0 to 10. Scores between 7 and 10 indicate good family functioning, scores between 4 and 6 indicate moderate impairment, while scores between 0 and 3 suggest severe impairment (17). The Cronbach's alpha coefficient for this scale was 0.773 (18), which has good reliability.

#### Toronto Alexithymia Scale (TAS-20)

The Toronto Alexithymia Scale is used to measure the level of dysarthria in patients. The scale consists of 20 entries on three dimensions: emotion recognition disorder, emotion expression disorder, and extroverted thinking. A 5-point Likert scale was used, with a total score ranging from 20 to 100. A score of <51 indicates the absence of Alexithymia, 51 to 61 is a critical Alexithymia, and a score of 61 and above indicates the presence of Alexithymia (19). The Cronbach's alpha coefficient for the scale in this study was 0.844 (20), good reliability and validity.

#### General Alienation Scale (GAS)

The General Alienation Scale was used to measure the level of social Alienation of patients. All items adopt the Likert 4 rating method with 4 dimensions, including self-alienation, other-alienation, skepticism, and meaninglessness. Each entry is assigned a score of 1 to 4, and the total score is 15–60, with higher scores implying a higher degree of social alienation (21). The Cronbachs alpha coefficient of the scale in this study was 0.816 (22), with good reliability.

### Data collection and quality control methods

Before writing the questionnaire for the research subjects, two postgraduate students with standardized training explained the study and the questionnaire content in detail and were collected by the researcher personally; in the process of filling out the questionnaire to the patients, who had doubts to give explanations and instructions at the right time; after the questionnaires were filled out by the research subjects, the questionnaires were checked for completeness, and if there were any omitted items or obvious logical errors, they were asked for their reasons to fill in the questionnaires, so as to ensure the accuracy of relevant information. Data processing was carried out with two-person entry and two-person verification to ensure the accuracy of data entry. The researcher distributed a total of 380 questionnaires, excluding questionnaires such as stopping to fill in halfway and errors, etc., and finally recovered 365 valid questionnaires, with an effective recovery rate of 96.1%.

### Statistical methods

Exploratory latent profile analysis was performed using Mplus 8.7 software. Patients with different scores of social alienation in older women with SUI were categorized, and the 15-item scores of the General Alienation Scale were used as exogenous indicators, and one to four profiles were selected sequentially for analysis. SPSS 27.0 statistical software was used for analysis. For measurement data that conformed to normal distribution, mean ± standard deviation was used; count data were expressed as constitutive ratios. Social factors and general demographic characteristics of patients in different social alienation categories were compared by chi-square test or one-way ANOVA. Variables showing statistical significance in the one-way analysis were used as independent variables, and the three potential categories of SUI in older women were used as dependent variables in a multivariate logistic regression analysis in order to explore the influencing factors, and a *P* < 0.05 was considered statistically significant. The model fit test indicators included (1) Aicheck information criterion (AIC), Bayesian information criterion (BIC), and sample-corrected Bayesian information criterion (aBIC), with smaller values indicating a better model fit; (2) Entropy of information (Entropy), which ranges from 0 to 1, with a value of >0.7 being a good fit, and nearer to 1 being a more accurate categorization; and (3) Romdale-Reuben corrected Likelihood Ratio Test (LMR) and Bootstrap Likelihood Ratio Test (BLRT), a *p* < 0.05 indicates that the kth model outperforms the k-1th model (16).

## Results

### General information social alienation in older female patients with SUI

A total of 380 questionnaires were distributed in this study, 365 questionnaires were recovered, and the recovery rate of valid questionnaires was 96.05%. The score of social detachment of older adult female patients with stress urinary incontinence was (39.08 ± 11.14), the score of dysphoria was (62.26 ± 9.54), and the score of family caring was (7.26 ± 2.49), and the general information of patients with SUI and different potential categories of Comparison of scores of SUI patients is shown in Table 1.

**Table 1:** Univariate analysis of social detachment in older adult female SUI patients (*N* = 365).

**Variable**		**Sum**	**C1**	**C2**	**C3**	**X^2^/F**	***P***
	60–70	236	74	125	37		
Age	71–80	110	26	48	36	15.481	0.004
	>80	19	7	6	6		
BMI	< 18.5	27	7	13	7	33.238	< 0.001
	18.5–23.9	171	65	90	16	
	24.0–27.9	123	28	55	40	
	≥28.0	44	7	21	16	
Education	Secondary schools or less	164	29	80	55	46.879	< 0.001
	Junior high school	132	42	68	22		
	High school	57	30	26	1		
	College and above	12	6	5	1		
Current address	City	197	79	96	22	38.699	< 0.001
	Village	168	28	83	57		
Living situation	Not live alone	303	102	156	45	51.701	< 0.001
	Live alone	62	5	23	34		
Marital status	Married	263	87	116	60	9.818	0.007
	Spouseless	102	20	63	19		
Professional status	Employed	26	15	10	1	67.890	< 0.001
	Separated or unemployed	181	24	92	65		
	Retired	158	68	77	13		
Monthly Income	< 1,500	123	27	68	28		
	1,500–2,999	149	34	75	40		
	2,999–5,000	76	33	32	11	30.857	< 0.001
	>5,000	17	13	4	0		
Other chronic diseases	0	132	71	56	5	113.057	< 0.001
	1–2	171	32	99	40		
	≥3	45	2	12	31		
	Unknown	17	2	12	3		
Alexithymia		62.26 ± 9.54	54.16 ± 9.43	64.10 ± 7.41	69.09 ± 5.75	*F* = 4.004	< 0.001
Family care		7.26 ± 2.49	8.46 ± 1.54	7.65 ± 2.15	4.76 ± 2.55	*F* = 19.906	< 0.001

### Results of a potential profile analysis of social alienation in older female patients with SUI

The results of this study showed that the social alienation scores of 365 older adult female SUI patients ranged from 15 to 60 (39.08 ± 11.14). Individual-centered latent profile analysis was performed on the social alienation scores of 365 older adult female SUI patients, and a total of 1–4 latent category models were fitted to the 15 entries of social alienation, as shown in Table 2. In the table, the AIC, BIC, and aBIC indexes decreased gradually with the increase of the number of categories, and the decrease decreased when it was changed from category 3–4, so category 3 could be chosen; according to the information entropy index, all models had the information entropy index, and the information entropy index had the information entropy index, and the information entropy index had the information entropy index, which was the same as the information entropy index. information entropy index, the information entropy values of all models are higher than the critical criterion of 0.8; and the results of BLRT and LMR significance indexes of the 3 models are significantly different (*P* < 0.001). Therefore, by combining the fitting index and practical significance of the model, three categories were finally selected as the optimal model for social alienation in older adult female SUI patients.

**Table 2:** Indicators of fit for a potential profile model of social alienation in order woman SUI (*n* = 365).

**Model**	**AIC**	**BIC**	**aBIC**	**Entropy**	**11p**	**14p**	**Categorical probability**
1	14,751.224	14,868.221	14,773.221	-	-	-	1
2	11,023.941	11,203.336	11,057.397	0.995	0.0000	0.0000	0.22/0.78
3	10,443.555	10,685.349	10,488.649	0.922	0.0000	0.0000	0.29/0.49/0.22
4	10,252.270	10,556.462	10,309.000	0.927	0.2278	0.0000	0.19/0.20/0.55/0.06

### Potential profiling and naming of social alienation in older adult female patients with stress urinary incontinence

**Figure 1:**
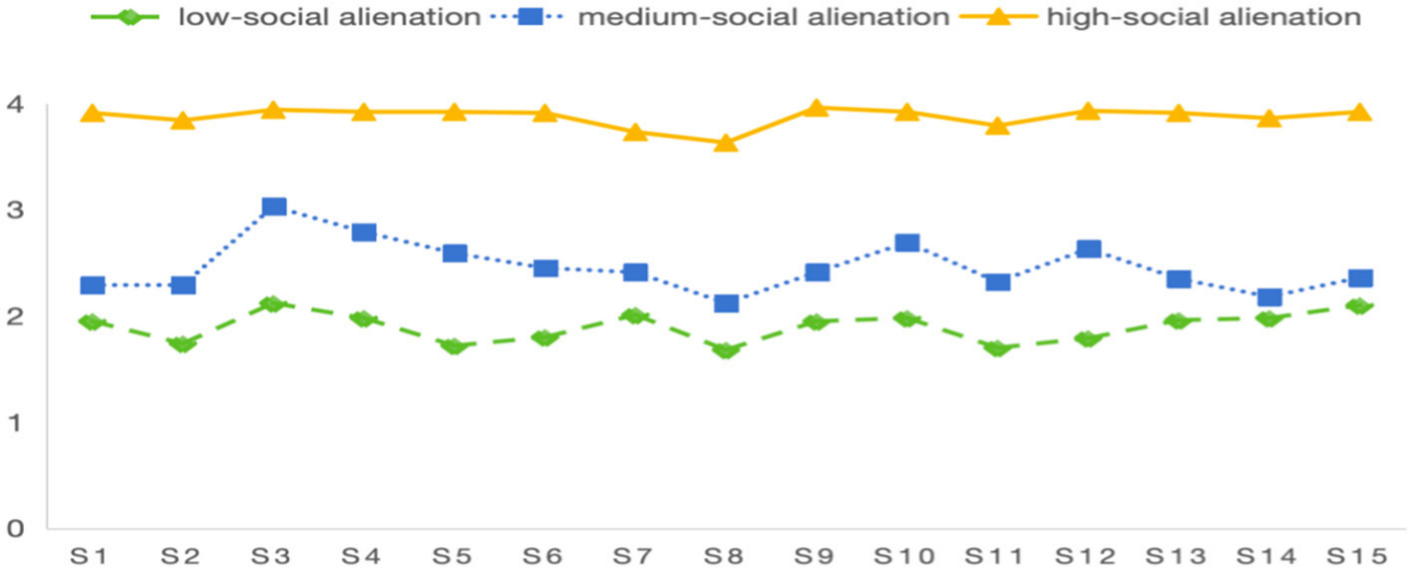
Distribution of three potential profile characteristic of social alienation in older adult women with SUI.

We analyzed the characteristics of three potential categories of social alienation in older adult women with SUI by drawing a potential profile of social alienation in older adult women with SUI (Figure 1), and named the three categories according to the characteristics of each potential category.

There were 107 cases (29.0%) in category 1 (low-social alienation) with a social alienation score of (28.51 ± 3.78), and the scores of each item were at a low level; there were 179 cases (49.4%) in category 2 (medium-social alienation) with a social alienation score of (36.98 ± 3.36), and the scores of the items of rest and sleep management were higher; and the scores of the items of category 3 (high-social alienation) and category 4 (high-social alienation) were at a high level.

-There were 79 cases (21.6%) in category 3 (high social alienation type), and the score of social alienation was (58.15 ± 1.49), and the scores of each item were at a high level.

### Results of a univariate analysis of potential profiles of social alienation in older adult women with stress urinary incontinence

The differences between the three potential categories of social alienation in SUI among older adult women were statistically significant (*P* < 0.05) in terms of age, BMI, educational level, occupational status, place of residence, mode of residence, marital status, per capita monthly household income, number of other chronic diseases combined, level of family care, and level of dysfunctional narratives, as shown in Table 1.

### Results of a multifactorial analysis of potential profiles of social alienation in older adult women with stress urinary incontinence

Multiple logistic regression analyses were performed using the three potential categories of social alienation in older adult women with SUI as dependent variables, family care and dysphoria as covariates, place of residence, BMI, mode of residence, occupational status, marital status, living alone or not, and combination of chronic diseases or not, as categorical variables, and the first category as the reference category. The results showed that narrative disorder, family care, city of residence, normal BMI, no other chronic disease, living alone, and being separated from work or unemployed were the factors influencing different potential categories of SUI in older women (all *P* < 0.05), see Table 3.

**Table 3:** Multiple Logistic Regression Analysis of Factors Influencing Potential Categories of Social alienation.

**Variable**	**C2**					**C3**				
	β	标准误	***P***	**OR**	**95%CI**	β	标准误	***P***	**OR**	**95%CI**
Intercept	−4.306	1.795	0.016	—	—	−15.329	3.463	< 0.001	—	—
Alexithymia	0.142	0.023	< 0.001	1.152	1.102–1.204	0.329	0.049	< 0.001	1.390	1.262 1.530
Family care	−0.157	0.089	0.079	0.854	0.717–1.018	−0.806	0.142	< 0.001	0.447	0.338 0.590
City	−0.242	0.364	0.507	0.785	0.385–1.603	−1.646	0.633	0.009	0.193	0.056 0.667
BMI normal	−0.633	0.582	0.277	0.531	0.170–1.662	−2.049	0.951	0.031	0.129	0.020 0.831
No other chronic	−2.811	0.922	0.002	0.06	0.01–0.367	−5.756	1.473	< 0.001	0.003	0.000 0.057
**Diseases**										
Live alone	0.493	0.665	0.458	1.638	0.445–6.034	2.057	0.839	0.014	7.823	1.511 40.502
Separated or unemployed	0.803	0.37	0.03	2.233	1.081–4.611	2.119	0.681	0.002	8.323	2.190 31.626
Spouseless	0.041	0.398	0.918	1.042	0.478–2.272	3.092	0.801	< 0.001	22.019	4.584 105.763

The study showed that living alone, the higher the probability of being categorized as high social alienation type (OR = 7.823, *P* = 0.014); being separated from work or unemployed, the higher the probability of being categorized as high social alienation type (OR = 8.323, *P* = 0.002); being without a spouse, the higher the probability of being categorized as high social alienation type (OR = 22.019, *P* < 0.001); and having higher scores of dysfunctional narratives, the higher the probability of being categorized as high social alienation type (OR = 1.390, *P* < 0.001); the higher the score for narrative disorder, the higher the probability of being categorized as high social alienation type (OR = 1.390, *P* < 0.001). The probability of being classified as highly socially alienated was lower for being from the city (OR = 0.193, *P* = 0.009); the probability of being classified as highly socially alienated was lower for not having other chronic diseases (OR = 0.003, *P* < 0.001); the probability of being classified as highly socially alienated was lower for higher scores of Family Care (OR = 0.447, *P* < 0.001); and the probability of being classified as highly socially alienated was higher for higher scores of Relational Difficulties (OR = 0.390, *P* < 0.001) . Alienation type (OR = 0.807, *P* < 0.001); the lower the score for dysphoria, the higher the probability of being categorized as high social alienation type (OR = 0.807, *P* < 0.001), see Table 3.

## Discussion

The social alienation scores of 365 older adult female SUI patients investigated in this study ranged from 15 to 60 (39.07 ± 11.14), and the results of this investigation were similar to those of Hong Xiuqin et al. (23), indicating that many SUI patients in China currently have different degrees of social alienation, and the reason for analyzing the results may be that frequent leakage of urine in SUI patients makes the patients more prone to inferiority complex, anxiety, depression, stigma and Loneliness and other negative emotions, due to the fear of suffering from discrimination and prejudice from others (24, 25), easy to gradually alienate the normal social network relationships, so that the patient's social willingness to reduce the social, social avoidance behavior increased, the emergence of social detachment. Patients' concerns about urine leakage significantly limit their participation in daily activities and social interactions, leading to social withdrawal and social isolation (26). The above findings suggest that the level of social detachment in older adult female patients with SUI needs to be reduced, and in view of the increasing number of patients with SUI, healthcare professionals should pay great attention to it and manage it effectively.

The results of this study showed that there was group heterogeneity in the social alienation of older adult female stress urinary incontinence patients, and the patients' social alienation could be categorized into three potential categories of low social alienation, medium social alienation-self-alienation, and high social alienation, and there was heterogeneity among the different categories. Analyzing the percentage of each category, the older adult SUI patients in the medium-high social alienation subgroup (high social alienation, medium social alienation-self-alienation) were 3.62 times higher than those in the low social alienation subgroup, suggesting that the overall social alienation of this group of patients was at a medium-high level. According to the responses of patients with medium social alienation-self-alienation type in individual entries, it was found that the scores of this group of patients were generally at a medium level, but the scores of the entries in the self-alienation dimension were all high, indicating that this group of patients had a series of psychological problems such as the sense of shame and loneliness caused by the disease, which led to their own initiative to alienate themselves from social relationships. Patients with high social alienation type SUI have high levels of social alienation in all scores of the entries and this type of patients have the worst avoidance and rejection of socialization, automatically and consciously alienating themselves from other people and the society, accompanied by serious negative emotions. Therefore, nursing care should strengthen the monitoring, assessment and analysis of social alienation level of SUI patients, to understand the psychological status of the patients and the problems in socialization, and to provide multifaceted health education and targeted intervention strategies to promote the participation of patients in social activities, etc., so as to reduce the level of social alienation in older adult female SUI patients, and to promote the physical and mental health of patients to recover.

The results of the present study found that the probability of being categorized as high social alienation type and medium social alienation type was high in nulliparous, and the level of social alienation was lower in spousal patients, which is consistent with the study of Cobo-Cuenc et al. (21), and may be due to the fact that women are more reliant on their families and socializing around them to obtain emotional support, while the nulliparous patients lacked the encouragement and urging of their families, and were highly prone to negative emotions, feeling lonely and The lack of encouragement and supervision from family members of patients without spouses makes them prone to negative emotions, feelings of loneliness and helplessness, and aggravates the deterioration of mental health. Patients with spouses can obtain more psychological and objective support, which can alleviate patients' negative emotions (27), thus reducing the level of social alienation. Therefore, healthcare professionals should pay attention to the marital status of patients and provide individualized psychological interventions according to the specific situation.

In this study, we found that patients who were employed had a higher probability of being categorized as the low social alienation type, which was similar to the findings of Shao Laojiao et al. (28). Compared with the older adult SUI patients who left their jobs or were jobless, the re-employed patients, who could find more of their own value from their jobs, communicated and interacted more closely with others, and had a good social adaptation, which was conducive to lowering the sense of social detachment. This suggests that in clinical nursing practice, more support and care should be given to the separated or jobless SUI patients, to channel their negative emotions and to guide them to cope with the disease positively.

The results of this study found that the higher the family caring score of older adult female SUI patients, the lower the probability of being categorized as high social detachment, and that high family caring is a protective factor for social detachment, which is consistent with the findings of Hua et al. (29). Good family functioning not only helps to reduce patients' negative emotions, but also helps patients to better adapt to the changes in family and social roles caused by the disease, increases patients' opportunities for interpersonal communication, and encourages patients to better express their emotions and thoughts, thus reducing the sense of social alienation (30, 31). Previous foreign studies on different populations (32) show that the level of social alienation of patients is closely related to their psychological and family functioning. The reason for this is that when patients have a high level of family care, their family function is relatively perfect, and patients can get more care and support from their families, which can avoid the generation of loneliness, depression and other negative emotions to a certain extent, and face the disease with a more positive mindset. It is recommended that healthcare professionals should pay special attention to the interaction of family care for SUI patients, make full use of the healthcare-family bridge, encourage family members to give adequate care and companionship to the patients, improve family functions, and create a warm and comfortable family atmosphere for them. This helps to accurately determine the patient's psychological state and level of positive mindset, so as to provide more personalized care services. For example, healthcare professionals can encourage additive members to actively participate in the patient's treatment process to increase the family's sense of knowledge, which in turn raises the patient's level of hope By nursing intervention in this way, it not only relieves the patient's specific symptoms, but also provides support for the patient at the psychological level to help them cope with the challenges of the disease in a better way.

The results of the present study indicated that patients living alone had a higher probability of being categorized as highly socially alienated relative to those living with their spouses or children. A related study (33) also confirmed that the reason for this may be that this group of patients have a significantly higher level of alienation due to the narrowing of their social circle, plus less care and comfort from family members. The present study found that low patients from rural areas had a higher probability of being categorized as high social alienation type, which is in line with Xiaohan et al. (34). The medical resources and economic level of rural areas are relatively poor, SUI patients may not be able to obtain timely and effective treatment, and the economic pressure is higher, at the same time, the rural areas are more conservative ideological concepts, there is a certain amount of discrimination and prejudice against SUI patients, leading to patients feel the sense of disease shame in the face of other people's dissimilarity, exacerbating the sense of helplessness in the face of the disease, which leads to the occurrence of social detachment.

The results of this study showed that BMI level is an influential factor in older adult SUI patients, and obesity will lead to SUI patients to be more likely to be discriminated against by others in the society and lack of friends, which will cause individuals to develop negative emotions such as low self-esteem, body imagery dissatisfaction, loneliness, and social avoidance, and exacerbate social barriers (30, 35), exulting in the more severe their social alienation. Consistent with the findings of Shiovitz-Ezra and Parag (36) suggests that medical personnel should pay attention to the mental health level of obese patients, and provide patients with weight loss-related mental health education and psychological adjustment skills support in a timely manner.

In addition, the present study found that patients with high levels of dysphoria were more likely to be categorized as having high levels of social isolation. Multiple studies have shown that dysphoria can predict and explain the level and causes of loneliness in individuals. That individuals with high levels of dysphoria lack the ability to perceive and describe emotions, leading to social blockages and failures (37), and that the gap with expectations increases their sense of social detachment (38). For the special group of SUI patients, the duration of the disease is long, the patient's long-term suppression of emotions and the site of the disease involves privacy, and it is difficult for the patient to express the disease and psychological distress to others, which increases the occurrence of dysphoria. When the level of dysphoria is high, patients lack the ability to express their inner emotions, and negative emotions cannot be eliminated in time or accumulated for a long time, which is very easy to produce negative emotions such as loneliness and depression. It is suggested that health care personnel can strengthen the attention to the patient's emotional cognitive situation, enrich spiritual activities, actively guide the patient's emotional recognition, care for emotional expression, and enhance the sense of meaning of their existence.

Healthcare professionals should screen high-risk SUI patients at an early stage, develop individualized interventions and guide them to early exercise to strengthen the function of pelvic floor muscles, so as to effectively reduce the incidence of SUI and mitigate the negative impact of SUI. On the one hand, the risk of developing SUI can be assessed, and health warnings can be issued to high-risk patients, so that interventions can be taken to reduce the likelihood of developing SUI or alleviate symptoms before they appear. On the other hand, patients with SUI can be closely monitored and adherence to early pelvic floor dysfunction exercises can be monitored. Most older adult patients do not know about their disease and need to strengthen health education. Medical personnel can improve the understanding and mastery of knowledge related to stress urinary incontinence among sick women in the community through small lectures, bulletin boards, public numbers, etc., so as to let this group of people understand the causes of SUI and effective treatments, to change the attitudes of the group toward medical treatment, and to alleviate the related symptoms of this group of people through active and effective treatments.

## Limitation

This study still has some limitation: first, the questionnaire method was used, and based on ethical principles, the subjects participated and cooperated voluntarily. In addition to recall bias, there may be self-selective bias, which may result in the incidence rate of adverse reactions being lower than the actual situation, and we should try to circumvent this problem in the future. Second, this study applied latent profile analysis to classify older adult female patients with stress urinary incontinence on an individual-centered basis and named them according to their respective characteristics, and the accuracy of the naming may need to be further investigated. In addition, the present study was a cross-sectional survey study that could not establish a causal relationship, and further longitudinal studies are needed to validate the findings. In addition, the sample coverage of this study was limited, only including patients from three communities in Jinzhou City, suggesting that a multicenter, large-sample survey could be conducted in the future to include more disease, family, and psychosocial factors to further validate and supplement potential categories of influencing factors and findings.

## Conclusion

Older adult women with SUI were categorized into three types of social alienation: low-social alienation, medium-social alienation, and high-social alienation. Older adult female SUI patients with different potential categories differed in terms of narrative disorders and family caring. The results of this study can help identify the heterogeneity of older adult female SUI patients, and help healthcare professionals to develop scientific and effective targeted intervention programs according to the characteristics of each category, to reduce the level of emotional dysfunction, to improve family care, to reduce the level of social alienation in older adult female patients with SUI, to alleviate the psychological pressure, to improve the effect of the intervention, and to ultimately improve the patient's health status and quality of life.

## Data Availability

The original contributions presented in the study are included in the article/supplementary material, further inquiries can be directed to the corresponding author.

## References

[1] Chi Z Bailong L Xiangfu Z. Progress in diagnosis and treatment of female stress urinary incontinence. Chin J Endourol. (2021) 15:537–41.

[2] Minassian VA Stewart WF Wood GC. Urinary incontinence in women: variation in prevalence estimates and risk factors. Obstet. Gynecol. (2008) 111:324–31. 10.1097/01.AOG.0000267220.48987.17 18238969

[3] Zhu L Lang J Liu C. The epidemiological study of women with urinary incontinence and risk factors for stress urinary incontinence in China. Menopause. (2009) 16:831–6. 10.1097/gme.0b013e3181967b5d 19240656

[4] Dan W Yue S Ying W. Psychological nursing in 80 patients of postpartum stress urinary incontinence in the analysis of application effect. J Int Psychiat. (2019) 46:189–92. 10.13479/j.cnki.jip.2019.01.056

[5] Wenhao B Licheng J. Advances in the diagnosis and treatment of female stress urinary incontinence. Shandong Medical J. (2022) 62:112–5.

[6] Pizzol D Demurtas J. Celotto S.Urinary incontinence and quality of life: a systematic review and meta-analysis. Aging Clin Exp Res. (2021) 33:25–35. 10.1007/s40520-020-01712-y 32964401 PMC7897623

[7] Lin Z Yuan Z. Review of studies on social alienation of vulnerable groups. J Changchun Univers Sci Technol. (2015) 28:45–50.

[8] Liang Y Hao G Wu M. Social isolation in adults with cancer: An evolutionary concept analysis. Front Psychol. 13:973640. 10.3389/fpsyg.2022.973640 36262430 PMC9574202

[9] Xiuqin H. Analysis of the current status and influencing factors of social alienation in middle-aged and elderly female urinary incontinence patients. Pract Clini Journal of Integrat Trad Chin West Med. (2023) 23:98–101. 10.13638/j.issn.1671-4040.2023.04.028

[10] Yaya Z Jiawen H Xiaoxuan B. Status quo and influencing factors of alexithymia in female patients with pelvic floor dysfunction. Chin Nurs Res. (2023) 37:554–9.

[11] Mengyu Z Lina G Liyuan G. Study on social isolation and its influencing factors in patients with stroke. Modern Prevent Med. (2023) 50:2051–2055+2061. 10.20043/j.cnki.MPM.202211258

[12] Kui Y Jian P Jun Z. The application of latent profile analysis in organizational behavior research. Adv Psychol Sci. (2020) 28:1056–70. 10.3724/SP.J.1042.2020.0105637113526

[13] Berlin KS Williams NA Parra GR. An introduction to latent variable mixture modeling (part 1): overview and cross-sectional latent class and latent profile analyses. J Pediatr Psychol. (2014) 39:174–87. 10.1093/jpepsy/jst084 24277769

[14] Yu WJ Ying LG Qing LX Liang CM Wang DN Li GJ at al. Resilience of infertile families undergoing in vitro fertilization: An application of the double ABC-X model. Appl Nurs Res. (2023) 69:151656. 10.1016/j.apnr.2022.151656 36635011

[15] Nambiar A K Arlandis S Bø K Cobussen-Boekhorst H Costantini E de Heide M at al. European association of urology guidelines on the diagnosis and management of female non-neurogenic lower urinary tract symptoms part 1: diagnostics, overactive bladder, stress urinary incontinence, and mixed urinary incontinence. Eur Urol. (2022) 82:49–59. 10.1016/j.eururo.2022.01.045 35216856

[16] Rumeng Q Qi Z Zhen S. Advances in the use of the ABC-X model in stress care management. J Nurs. (2024) 31:47–51. 10.7748/nm.31.2.19.s8

[17] Smilkstein G Ashworth C Montano D. Validity and reliability of the family apgar as a test of family function. J Fam Pract. (1982) 15:303–11. 7097168

[18] Fan L Guang Z Songnuan L. A study on validity and reliability of the amily APGAR. Chin J Public Health. (1999) 15:987–988.

[19] Bagby R M Parker J D A Taylor G J. The twenty-item Toronto Alexithymia scale—I. item selection and cross-validation of the factor structure. J Psychosomatic Research. (1994) 38:23–32. 10.1016/0022-3999(94)90005-1 8126686

[20] Jinyao Y Shuqiao Z Xiongzhao Z. The Chinese version of the TAS-20: reliability and validity. Chin Ment Health J. (2003) (11):763–7.

[21] Cobo-Cuenca AI Martín-Espinosa NM Rodríguez-Borrego MA Carmona-Torres JM. Determinants of satisfaction with life and self-esteem in women with breast cancer. Qual Life Res. 28:379–387. 10.1007/s11136-018-2017-y 30324585

[22] Xiaoting W Aiqin C Hailing Z Yan J. Reliability and validity of the generalized social of alienation scale among the elderly. J Chengdu Med College. (2015) 10:751–4.

[23] Xiuqin H. Analysis of the current status and influencing factors of social alienation in middle-aged and elderly female urinary incontinence patients. Pract Clini J Integrat Trad Chin Western Med. (2023) 23:98–101.

[24] Zhang X Ma L Li J Zhang W Xie Y Wang Y at al. Mental health and lower urinary tract symptoms: Results from the NHANES and Mendelian randomization study. J Psychosom Res. (2024) 178:111599. 10.1016/j.jpsychores.2024.111599 38309129

[25] Cox CK Schimpf MO Berger MB. Stigma associated with pelvic floor disorders. Female Pelvic Med Reconstr Surg. (2021) 27:961. 10.1097/SPV.0000000000000961 33105346

[26] Rodriguez-Almagro J Hernandez Martinez A Martinez-Vazquez S. A qualitative exploration of the perceptions of women living with pelvic floor disorders and factors related to quality of life. J Clin Med. (2024) 13:1896. 10.3390/jcm13071896 38610661 PMC11012559

[27] Yi Z Yongxia M Zhiwei L Zhenxiang Z Yipu S Beilei L. Analysis of the subject-object interdependence model of family resilience on the fear of progression in stroke patients and their spousal caregivers. J Nurs Sci. (2024) 39:78–81.

[28] Lijiao S Junxia W Tianrui W Yuhua Z Shuyi G Ruixing Z. Analysis of the current situation of social alienation and influencing factors of patients with intestinal stoma. J Nurs Sci. (2022) 29:19–23. 10.16460/j.issn1008-9969.2022.15.019

[29] Hua C Honghui S Liang L Bo Z Caihong Z. Mediation effect of family functioning and psychological resilience between stigma and social alienation among patients with flap transplantation. Milit Nurs. (2024) 41:33–36+40.

[30] Shi H Cuiping T Qi W Hongbin W Xingwei Z. Research progress of social isolation in patients with cardiovascular disease. Health Res. (2024) 44:430–4. 10.19890/j.cnki.issn1674-6449.2024.04.015

[31] Honglun W Yingxiang Z Haiyan W Mei T Xiaoying Y. Current status and influencing factors of social isolation in elderly diabetic patients. Chin J Nurs Educ. (2023) 20:594–8.

[32] Yi ES Hwang HJ A. study on the social behavior and social isolation of the elderly Korea. J Exerc Rehabilitat. (2015) 11:125–32. 10.12965/jer.150215 26171377 PMC4492421

[33] Yinhu C Jingjie M. Influence of social participation on mental health of the elderly living alone based on propensity score matching. Med Soc. (2023) 36:69–73. 10.13723/j.yxysh.2023.02.013

[34] Xiaohan G Wanli M Dongyan W Jinlei B Shujuan L. A study of social alienation in maintenance hemodialysis patients. J Nurs Sci. (2024) 39:79–82.

[35] Ruiqi G Yi H Shuhui M Xiaofen C Bei L. Characteristics of depression categories and their relationship with sleep in overweight and obese adolescents based on latent profle analysis. Modern Prevent Med. (2022) 49:4490–4494+4536. 10.20043/j.cnki.MPM.202205171

[36] Shiovitz-Ezra S Parag O. Does loneliness ‘get under the skin'? Associations of loneliness with subsequent change in inflammatory and metabolic markers. Aging Ment Health. (2019) *Aging Ment Health*. 23(10):1358–1366. 10.1080/13607863.2018.1488942 30380911

[37] Xiaoting W Aiqin C Hailing Z Yan J. Mediating effect of loneliness between alexithymia and depression in elderly patients withchronicconditions in the community. Chin General Pract. (2021) 24:4563–8.

[38] Morr M Lieberz J Dobbelstein M Philipsen A Hurlemann R Scheele D. Insula reactivity mediates subjective isolation stress in alexithymia. Scientific Reports. (2021) 11:15326. 10.1038/s41598-021-94799-w 34321519 PMC8319294

